# Interaction between baseline HBV loads and the prognosis of patients with HCC receiving anti-PD-1 in combination with antiangiogenic therapy undergoing concurrent TAF prophylaxis

**DOI:** 10.1186/s12879-022-07602-0

**Published:** 2022-07-14

**Authors:** Xiaoyun Hu, Rong Li, Qi Li, Mengya Zang, Guosheng Yuan, Jinzhang Chen

**Affiliations:** Department of Infectious Diseases and Hepatology Unit, Nanfang Hospital, Southern Medical University, 1,838 North Guangzhou Ave, Guangzhou, 510515 Guangdong China

**Keywords:** Hepatocellular carcinoma (HCC), Hepatitis B virus (HBV), Programmed cell death-1 (PD-1)/programmed cell death-ligand 1 (PD-L1), Reactivation, Tenofovir alafenamide fumarate (TAF)

## Abstract

**Background:**

A high baseline hepatitis B virus (HBV) load has always been listed as an exclusion criterion for programmed cell death-1 (PD-1) inhibitor-associated therapy in clinical trials, as the interaction between HBV load and anti-PD-1/PD-L1 therapy with anti HBV therapy remains controversial.

**Methods:**

We retrospectively enrolled 70 unresectable HCC patients who were seropositive for HBsAg and accepted tenofovir alafenamide fumarate (TAF) therapy before anti-PD-1 in combination with an antiangiogenic treatment. Patients were divided into a low HBV DNA group (≤ 2000 IU/ml) and a high HBV DNA group (> 2000 IU/ml) according to the baseline HBV DNA levels. Tumour response and progression-free survival (PFS) were compared, and univariate and multivariate Cox analyses were performed to identify potential risk factors for PFS. The incidences of HBV reactivation and HBV-associated hepatitis were also recorded.

**Results:**

48 patients were assigned to the low group and the remaining 22 patients were assigned to the high group. The objective response rates (ORRs), disease control rates (DCRs), and PFS between the two groups showed no significant difference (*P* = 0.761, 0.552, and 0.784, respectively). The results of Cox analyses revealed that there was no relationship between baseline HBV load and PFS. Additionally, HBV reactivation occurred in only 2 patients (2.9%), and no patient experienced HBV-related hepatic impairment when given a continuous TAF treatment.

**Conclusions:**

Baseline HBV loads do not affect the prognosis of HCC patients receiving anti-PD-1 in combination with an antiangiogenic therapy, while PD-1 inhibitors do not aggravate HBV reactivation and hepatic impairment in patients simultaneously subjected to TAF prophylaxis.

## Key points

Baseline HBV loads do not affect the prognosis of HCC patients receiving anti-PD-1 in combination with antiangiogenic therapy. Besides, PD-1 inhibitors do not aggravate HBV reactivation and hepatic impairment undergoing concurrent TAF prophylaxis.

## Introduction

Despite the existence of an effective hepatitis B virus (HBV) vaccine and antiviral therapies, HBV-related HCC remains a leading cause of death worldwide, particularly in Asia and Africa [1–3]. More than 350 million people are chronically infected with HBV worldwide [4, 5]. Unfortunately, over 70% of HBV-HCC cases are diagnosed at a late stage, which results in limited treatment options and poor prognosis [6]. Recently, immune checkpoint inhibitors (ICIs) like antibodies for programmed cell death-1 (PD-1), programmed cell death-ligand 1 (PD-L1) and cytotoxic T lymphocyte-associated antigen 4 (CTLA-4), have been reported to be the hotspot of HCC immunotherapy, since the interaction between PD-1, CTLA-4, and their ligands (PD-L1 and CD80/86) inhibits T-cell activation. For advanced HCC patients, PD-1/PD-L1 pathway inhibitor in combination with an antiangiogenic therapy has been demonstrated to be an effective treatment regimen [7–10]. However, a high baseline HBV DNA level has always been listed as an exclusion criterion for PD-1/PD-L1 inhibitor-associated therapy in clinical trials, regardless of the antiviral strategies. This is due to the controversial nature of the interaction between HBV load and anti-PD-1/PD-L1 therapy, particularly in HCC patients.

Several studies have shown that HBV reactivation induced by immunosuppressive agents or cytotoxic chemotherapy is a complication in cancer patients with pre-existing HBV infection, especially in those not subjected to continuous antiviral therapy [11–13]. Furthermore, Zhang et al. [14] demonstrated that the absence of antiviral prophylaxis treatment was the only significant risk factor for HBV reactivation following anti-PD-1/PD-L1 immunotherapy. Tenofovir alafenamide fumarate(TAF),a novel pro-drug of tenofovir (TFV) that has been approved for the treatment of chronic HBV infection, is characterized by a greater plasma stability and higher renal safety than tenofovir disoproxil fumarate (TDF) [15, 16].

However, to the best of our knowledge, no study has investigated the effects of anti-PD-1 treatment in combination with an antiangiogenic therapy on HBV infection in TAF prophylaxis-subjected individuals. Hence, we conducted a retrospective study to explore the effects of HBV load on anti-PD-1 in combination with an antiangiogenic therapy and the rate of HBV reactivation and hepatitis during a combined anti-PD-1 and antiangiogenic treatment.

## Methods

### Study design and patients

**Fig. 1:**
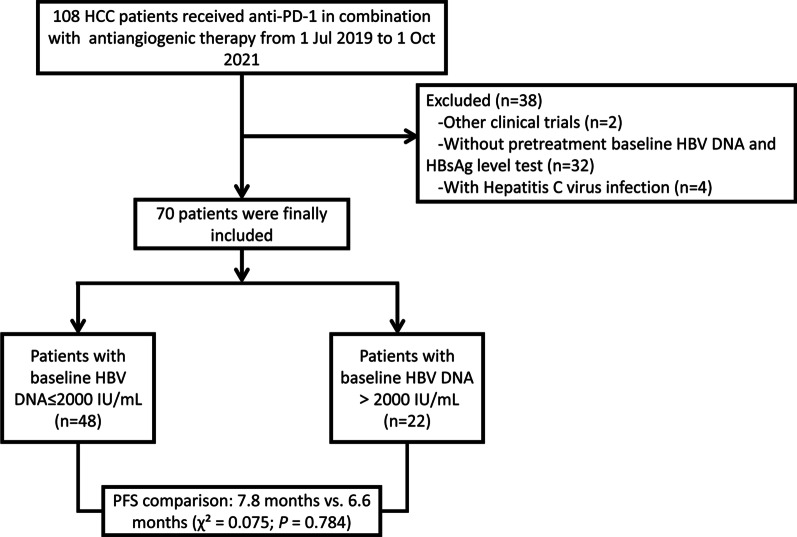
Flowchart of patient selection

We performed a retrospective cohort study on unresectable HCC patients who were seropositive for HBsAg and accepted TAF therapy prior to combined anti-PD-1 and antiangiogenic treatment. This study included consecutive patients who were referred to Nanfang Hospital, Southern Medical University in Guangzhou, China, between Jul 2019 and Oct 2021. A total of 108 patients were screened for eligibility according to the following inclusion criteria: (1) pathologically diagnosed with HCC; (2) received at least one cycle of anti-PD-1 therapy; (3) seropositive for HBsAg and had received TAF therapy as a regular antiviral regimen before anti-PD-1 treatment; and (4) with HBV DNA and liver function monitored regularly during the follow-up. Patients were excluded for: (1) the presence of other positive viral markers, including IgM antibodies to the hepatitis A virus, hepatitis C virus (HCV), or hepatitis E virus, IgG antibodies to the hepatitis D virus or HIV; (2) participating in other clinical trials; and (3) not having baseline HBV DNA and HBsAg test results (baseline defined as within 2 weeks prior to initial PD-1 inhibitor therapy). Finally, 70 patients with complete data were included in the current study. Figure 1 shows a flowchart of the patient selection procedure. This study was performed in accordance with the ethical guidelines of the 1975 Declaration of Helsinki and was approved by the ethics committee of the Nanfang Hospital, Southern Medical University. Informed consent was obtained from all the patients prior to their participation.

### Clinical endpoints

Eligible patients were divided into a low HBV DNA group (low group, ≤ 2000 IU/ml) and a high HBV DNA group (high group, > 2000 IU/ml) according to the baseline HBV DNA levels. The primary study end point was progression-free survival (PFS), which was defined as the time from the first dose of anti-PD-1 immunotherapy to the first radiological disease progression or death according to the Response Evaluation Criteria in Solid Tumours (RECIST) criteria, version 1.1. The secondary endpoints included HBV reactivation and HBV-associated hepatitis. HBV reactivation in patients positive for HBsAg was defined according to the American Association for the Study of Liver Diseases 2018 Hepatitis B guidance: (1) a ≥ 2 log (100-fold) increase in HBV DNA compared to the baseline level; (2) HBV DNA ≥ 1000 IU/mL in a patient with a previously undetectable level [17]. HBV-associated hepatitis was defined as a three-fold or greater increase in serum ALT or AST than the upper limit of a normal value or an absolute increase in serum ALT to more than 100 U/L accompanying or following HBV reactivation [18]. Follow-up computed tomography (CT) or magnetic resonance imaging (MRI) scans were performed 6–12 weeks after anti-PD1 treatment initiation and approximately 3–6 months thereafter. Serum HBV DNA level was measured with the Cobras Taqman HBV Kit (Roche Diagnostics; lower limit of detection: 20 IU/mL) and was also tested at each follow-up visit.

### Statistical analysis

All statistical analyses were performed using the SPSS (version 25, Chicago, NY, USA) and R software (version 3.6.2, http://www.Rproject.org). Data were expressed as counts (percentages) for categorical variables, as mean ± standard deviation (SD) for quantitative variables according to normal distribution, and as median (range) for variables with non-normal distribution. Pearson χ^2^ tests or Fisher’s exact tests were used to analyse relationships between categorical variables. PFS was performed using the Kaplan–Meier method. Univariate analyses were performed with the log-rank test, and variables with a *P* value lower than 0.1 were included in the multivariate analysis. Multivariate analyses were performed using the Cox’s proportional hazard regression model. Statistical significance was set at *P* < 0.05.

## Results

### Patients’ characteristics

The clinical characteristics of the 70 patients with HCC are summarised in Table 1. Accordingly, the mean age was 52.5 ± 12.2, and 66 (94.3%) patients were male. Regarding relative tumour features, 69 patients (98.5%) had an Eastern Cooperative Oncology Group (ECOG) performance status of 0 or 1; 42 (60.0%) had a portal vein tumour thrombus (PVTT); 25 (35.7%) had extrahepatic metastasis; 46 (65.7%) had more than 3 tumour lesions; 33 (47.1%) had the largest tumour diameter of over 7 cm; and 30 (47.1%) had an AFP level > 400 ng/mL. All patients received anti-PD-1 in combination with an antiangiogenic therapy, and the antiangiogenic regimens included lenvatinib (58.6%), sorafenib (11.4%), apatinib (7.2%), regorafenib (11.4%), and bevacizumab (11.4%). At baseline, 48 patients (68.6%) had a low HBV DNA level (baseline viral load ≤ 2,000 IU/mL), while 22 patients (31.4%) had a high HBV DNA level (baseline viral load > 2,000 IU/mL). In addition, the median HBsAg level was 403.9 (0.1, 7026.0) and 87.1% of patients were HBeAg-negative.

**Table 1:** Baseline characteristics of the 70 HCC patients receiving anti-PD-1 in combination with antiangiogenic therapy

Characteristics	All patients (n = 70)
Gender	
Male, n (%)	66, (94.3)
Female, n (%)	4, (5.7)
Age (y)^Δ^	52.5 ± 12.2
BCLC stage	
B, n (%)	18, (25.7)
C, n (%)	52, (74.3)
Child–Pugh class	
A, n (%)	55, (78.6)
B, n (%)	15, (21.4)
ECOG performance	
0, n (%)	50, (71.4)
1, n (%)	19, (27.1)
2, n (%)	1, (1.4)
Portal vein tumor thrombus	
Yes, n (%)	42, (60.0)
No, n (%)	28, (40.0)
Extrahepatic metastasis	
Yes, n (%)	25, (35.7)
No, n (%)	45, (64.3)
Tumor number	
< 3, n (%)	24, (34.3)
≥ 3, n (%)	46, (65.7)
Largest tumor diameter	
< 7 cm, n (%)	37, (52.9)
≥ 7 cm, n (%)	33, (47.1)
α-Fetoprotein level	
< 400 ng/mL, n (%)	40, (57.1)
≥ 400 ng/mL, n (%)	30, (42.9)
Antiangiogenic drug	
Lenvatinib, n (%)	41, (58.6)
Sorafenib, n (%)	8, (11.4)
Apatinib, n (%)	5, (7.2)
Regorafenib, n (%)	8, (11.4)
Bevacizumab, n (%)	8, (11.4)
ALT (U/L)*	31.5 (9.0, 146.0)
AST (U/L)*	46.0 (14.0, 225.0)
Albumin (g/L)^Δ^	36.7 ± 4.7
Total bilirubin (mmol/L)*	15.2 (5.2, 55.8)
PLT (10^9^/L)*	151.5 (34.0, 695.0)
PT (s)^Δ^	11.7 ± 1.3
HBV DNA	
≤ 2000 IU/mL, n (%)	48, (68.6)
> 2000 IU/mL, n (%)	22, (31.4)
HBsAg (IU/mL)*	403.9 (0.1, 7026.0)
HBeAg	
Positive, n (%)	9, (12.9)
Negative, n (%)	61, (87.1)

*BCLC* Barcelona-clinic liver cancer, *ECOG* Eastern cooperative oncology group, *ALT* alanine aminotransferase, *AST* aspartate aminotransferase, *PLT* platelet count, *PT* prothrombin time

^Δ^Normal distribution (mean ± Standard deviation); *non-normal distribution [median, (minimum, maximum)]

### Tumour responses

Tumour responses are shown in Table 2. Of all the included patients, 1 achieved a complete response (CR) (1.4%), 14 achieved a partial response (PR) (20.0%), and 36 patients had a stable disease (SD) (51.4%), resulting in an objective response rate (ORR) of 21.4% and a disease control rate (DCR) of 72.9%. The subgroup analysis revealed that ORRs in patients with low and high baseline HBV DNA levels were 22.9% and 18.2%, respectively (Fisher’s exact test, *P* = 0.761), while the DCRs were 75.0% and 68.2%, respectively (χ^2^ = 0.355; *P* = 0.552).

**Table 2:** Best tumor responses of patients with low and high baseline HBV DNA level

Tumor response	All patients (n = 70)	Baseline HBV DNA ≤ 2000 IU/mL (n = 48)	Baseline HBV DNA > 2000 IU/mL (n = 22)
Complete response (CR)	1 (1.4)	1 (2.1)	0
Partial response (PR)	14 (20.0)	10 (20.8)	4 (18.2)
Stable disease (SD)	36 (51.4)	25 (52.1)	11 (50.0)
Progressive disease (PD)	19 (27.1)	12 (25.0)	7 (31.8)
ORR (CR + PR)^Δ^	15 (21.4)	11 (22.9)	4 (18.2)
DCR (CR + PR + SD)*	61 (72.9)	36 (75.0)	15 (68.2)

*ORR* objective response rate, *DCR* disease control rate

^Δ^Fisher’s exact test, *P* = 0.761; *Pearson χ^2^ = 0.355, *P* = 0.552

### Baseline HBV loads do not affect PFS

**Fig. 2:**
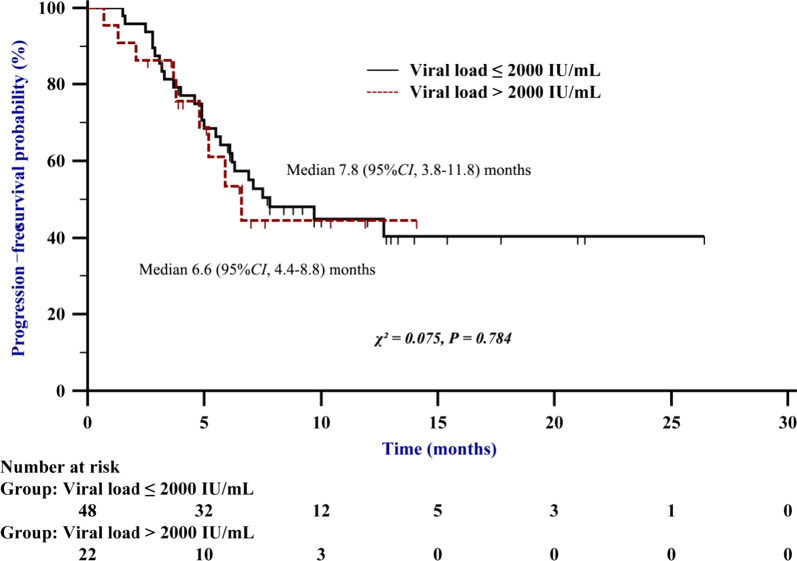
The progression-free survial (PFS) curves of patients with baseline HBV DNA level ≤ 2000 IU/mL and those with DNA level > 2000 IU/mL receiving anti-PD-1 in combination with antiangiogenic therapy

**Fig. 3:**
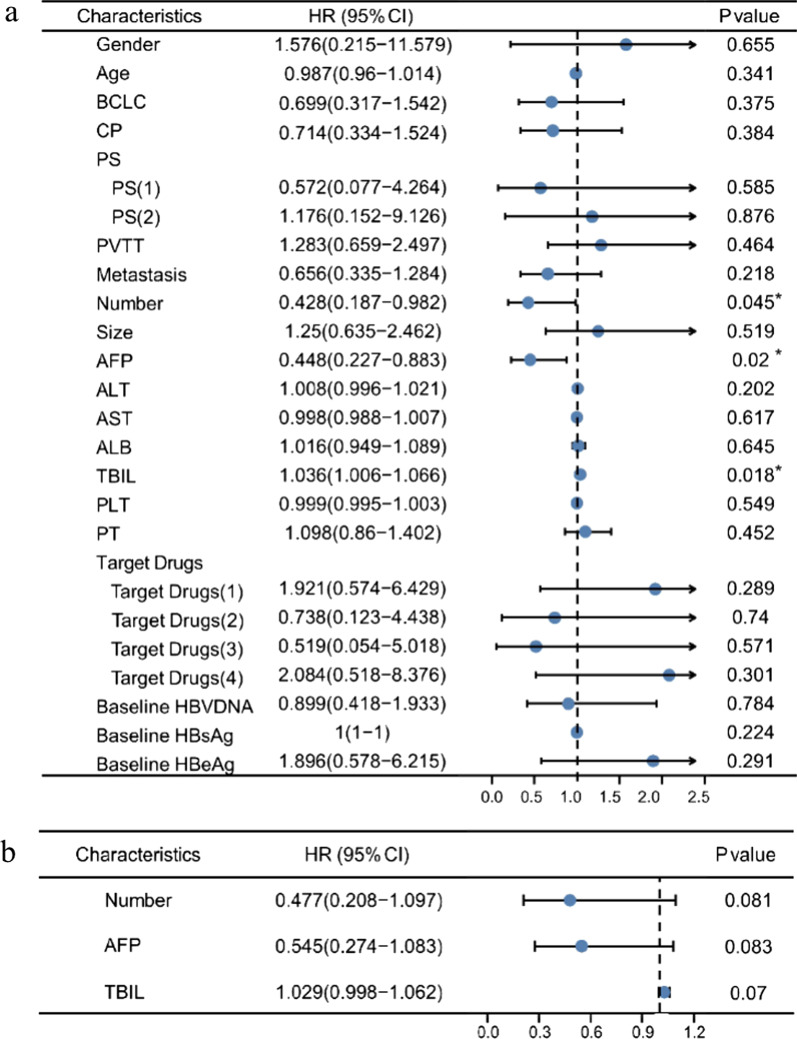
Univariate and multivariate cox analyses of progression-free survial (PFS) stratified by baseline characteristics

During treatment, 35 patients (50.0%) had a progressive disease. The median PFS time was 7.5 months. There was no significant difference in PFS between patients with a baseline HBV DNA level ≤ 2000 IU/mL and those with a DNA level > 2000 IU/mL (χ^2^ = 0.075; *P* = 0.784) (Fig. 2). To further identify whether baseline variables, especially HBV loads, affect PFS, univariate and multivariate Cox analyses were conducted. Univariate Cox regression analysis identified the following factors that affected PFS: tumour lesion number < 3 (OR 0.428, 95% CI, 0.187–0.982; *P* = 0.045), AFP level < 400 ng/mL (OR 0.448, 95% CI, 0.227–0.883; *P* = 0.020), and TBIL (OR 1.036, 95% CI, 1.006–1.066; *P* = 0.018). We then included these significant factors in a multivariate analysis, but found no independent predictive factors that affected PFS (Fig. 3).

### HBV reactivation and hepatitis

**Fig. 4:**
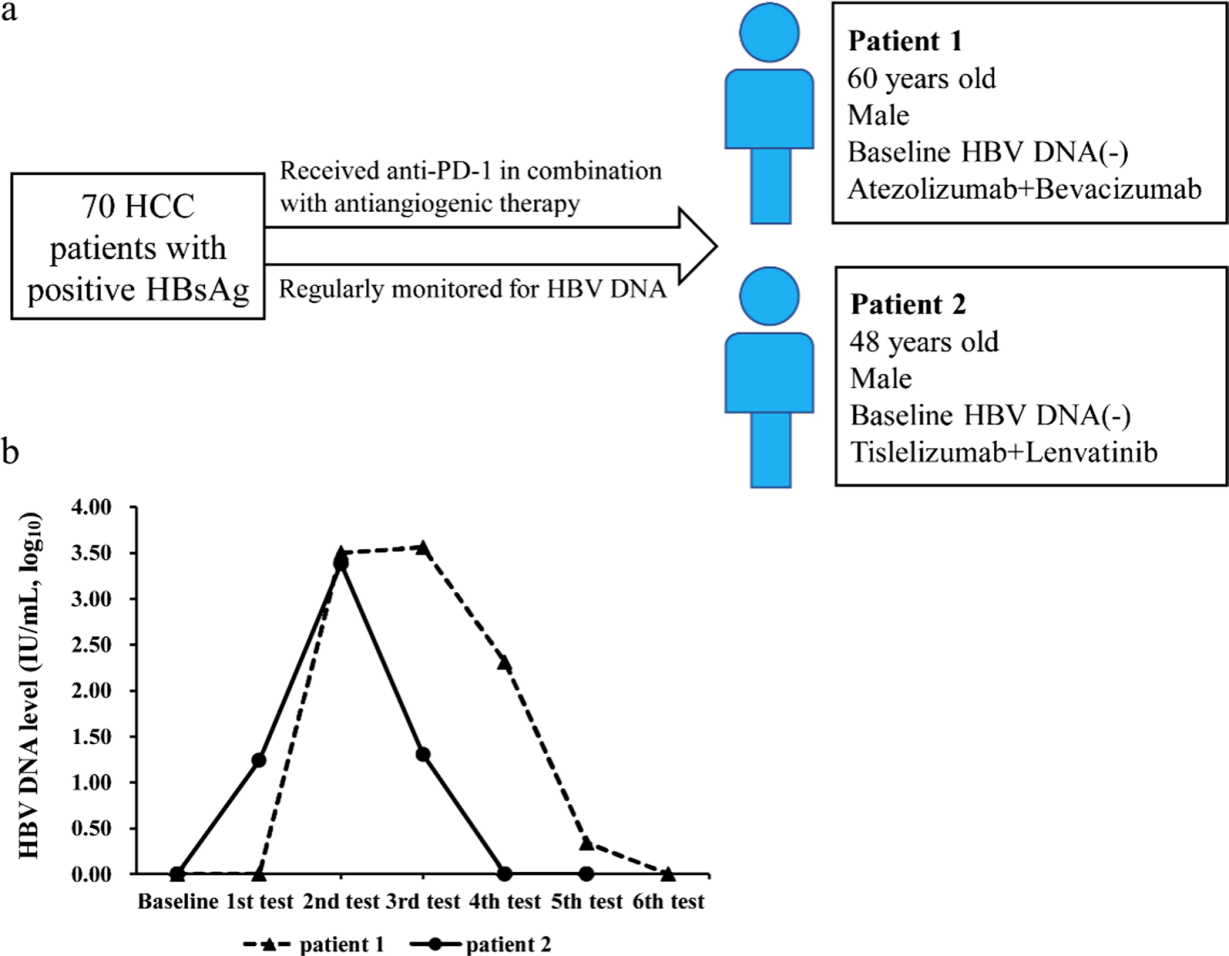
Among the 70 patients, 2 had HBV reactivation. **a** Characteristics of the 2 patients with HBV reactivation; **b** Kinetics of HBV DNA in the 2 patients with HBV reactivation

During the follow-up period, only two of the 70 patients (2.9%) experienced HBV reactivation. The details of the 2 patients with HBV reactivation are shown in Fig. 4. Briefly, both were male and had undetectable baseline HBV DNA levels. Patient 1 received atezolizumab in combination with bevacizumab and developed HBV reactivation at the 2nd visit (after 5 doses of atezolizumab). The patient’s HBV DNA level was 3162.3 IU/mL at diagnosis of HBV reactivation, achieved the highest level at the 3rd visit (3622.8 IU/ML), but fell to 205.1 IU/mL, < 10 IU/mL and undetectable at the 4th, 5th and 6th visit, respectively. The peak ALT level was 68 U/L during the follow-up. Patient 2 received tislelizumab in combination with lenvatinib and developed HBV reactivation at the 2nd visit (after 6 doses of tislelizumab). The patient’s HBV DNA level was 2417 IU/mL at diagnosis of HBV reactivation (the highest level), but fell to 20.2 IU/mL and undetectable at the 4th and 5th visit, respectively, with a peak ALT level of 49 U/L. Accordingly, both patients 1 and 2 exhibited a brief increase in HBV DNA levels without HBV-associated ALT elevation. None of the patients experienced immunotherapy disruption during the follow-up. In addition, 16 of the 70 patients (22.9%) experienced ALT elevation; however, all of these were considered cases of immune-related hepatitis as none of the 16 patients suffered from HBV reactivation.

## Discussion

To date, it has been confirmed that the HBV DNA level is positively correlated with the up-regulated expression of PD-1 on T cells, which is closely linked to the formation of HCC immunosuppressive microenvironment [19, 20]. The PD-1/PD-L1 axis also plays an important role in HBV replication [21]. Recently, the combination regimens of PD-1/PD-L1 inhibitor and an antiangiogenic therapy have been proven to be an optimal treatment for advanced HCC [7, 8, 22, 23]. However, the interaction between HBV load and anti-PD-1/PD-L1 therapy remains controversial, particularly in patients who do not receive a continuous antiviral therapy. Several studies have reported high HBV load as a risk factor for HBV reactivation and hepatic impairment during anti-PD-1/PD-L1 therapy [24, 25], while other reports demonstrated that PD-1/PD-L1 inhibitors can be safe and effective in cancer patients with either chronic HBV or HCV infection [26, 27]. Different antiviral agents, such as lamivudine (LAM) or adefovir dipivoxil (ADV), may account for the controversial results in studies reporting HBV load to be a risk factor for HBV reactivation. However, there is no data demonstrating this issue, since the most commonly used agent was entecavir (ETV) or tenofovir (TDF) in the currently reported studies, where LAM and ADV were no longer recommended as first-line anti-viral regimens. Most of the relevant studies did not explore whether antiviral therapy can improve the efficacy and safety of anti-PD-1 treatment in combination with an antiangiogenic therapy. In the current study, we found that baseline HBV load did not affect the prognosis of HCC patients receiving anti-PD-1 combined with an antiangiogenic therapy, while PD-1 inhibitors did not aggravate HBV reactivation and hepatic impairment in patients given TAF prophylaxis.

Since evidence regarding whether HBV infection affects the prognosis of HCC patients receiving anti-PD-1 based therapy is scarce, patients with a high baseline HBV DNA level were always excluded from clinical trials regardless of the antiviral strategies utilized, limiting their efficiency and generalizability. In the KETNOTE-224 study, tumour response was comparable between patients with and without HBV/HCV infection [27]. Similarly, the CheckMate 040 study reported similar tumour responses among patients with advanced HCC, irrespective of HCC aetiology [26]. However, patients with a higher baseline HBV DNA level (usually > 500 IU/mL or > 2000 IU/mL) were excluded, and whether baseline HBV DNA level affected the clinical prognosis of HCC patients receiving anti-PD-1 based therapy was not assessed in the above clinical trials. In a retrospective study in China, the baseline HBV load was found to have no significant impact on the prognostic outcomes or rates of hepatic impairment during anti-PD-1 blockade [28]. According to our results, similar ORR and DCR were observed in patients with low and high baseline HBV DNA levels. In addition, there was no significant difference in PFS between patients with a higher or lower baseline HBV loads. Importantly, our data highlight that HBV load may not affect the prognosis of HCC patients receiving anti-PD-1 therapy combined with an antiangiogenic therapy.

Whether PD-1 inhibitors aggravate HBV reactivation and hepatic impairment is another concern of anti-PD-1-based therapies. In a phase Ib study comparing nivolumab with and without an HBV therapeutic vaccine, in virally suppressed patients with HBeAg (−) chronic HBV, PD-1 inhibitor was demonstrated to be well tolerated and led to HBsAg decline in most patients [29]. In a study comparing HBV reactivation between patients with low and high HBV DNA loads, who were undergoing anti-PD-1 blockade treatment, similar incidences of HBV reactivation and HBV-associated hepatitis were observed [30]. In the current study, only 2 of the 70 patients (2.9%) experienced HBV reactivation, which was a lower rate compared to patients with other cancer types in another study [14]. The reason for this discrepancy may be that all patients in our study simultaneously received TAF prophylaxis. Continuous and effective antiviral treatment was shown to improve the prognosis of HCC patients receiving anti-PD-1 blockade with high viral loads in our previous study (recently accepted article, https://doi.org/10.21037/atm-21-3020). Nevertheless, the specific role of TAF in the protection against HBV reactivation or hepatic impairment has not been elucidated, since TAF has been proven to have a greater plasma stability and higher renal safety than TDF. In addition, we did not observe any cases of HBV-related hepatic impairment during the follow-up period. Taken together, we suggest that HBV-HCC patients accept first-line antiviral prophylaxis such as TAF before and during the period of anti-PD-1-based therapy.

The current study is not free from certain limitations. First, this single-arm study was designed retrospectively, which may have caused bias in the selection of patients. The implications of this study need to be verified by future clinical studies with larger sample sizes. Second, the overall survival (OS) data were not included in the analysis, as the follow-up period was not long enough, and only two patients died until the observation deadline. Finally, patients with HCV infection were excluded from the final analysis, and the influence of HCV loads on these patients remains unclear.

In conclusion, our study provides evidence that baseline HBV loads do not affect the prognosis of HCC patients receiving anti-PD-1 in combination with antiangiogenic therapy, while PD-1 inhibitors do not aggravate HBV reactivation and hepatic impairment in patients given TAF prophylaxis. However, as this was a non-randomized retrospective study, our data should not be taken as non-biased or used to guide clinical decisions without a further proof derived from prospective clinical trials. Besides, future prospective studies should also pay attention to the interaction between HBV reactivation and the combination therapy of anti-PD-1 and anti-CTLA-4, since trials are ongoing to explore the possibility of CTLA-4 in combination with anti-PD-1/PD-L1.

## Data Availability

The data that support the findings of this study are available from the corresponding author upon reasonable request.

## Abbreviations

HBV: Hepatitis B virus
PD-1: Programmed cell death-1
TAF: Tenofovir alafenamide fumarate
PFS: Progression-free survival
ORRs: Objective response rates
DCRs: Disease control rates
PD-L1: Programmed cell death-ligand 1
TDF: Tenofovir disoproxil fumarate
HCV: Hepatitis C virus
RECIST: Response Evaluation Criteria in Solid Tumours
CT: Computed tomography
MRI: Magnetic resonance imaging
ECOG: Eastern Cooperative Oncology Group
PVTT: Portal vein tumour thrombus
CR: Complete response
PR: Partial response
SD: Stable disease
OS: Overall survival
